# Separation of Monocytes from Whole Human Blood

**DOI:** 10.1100/tsw.2002.842

**Published:** 2002-06-07

**Authors:** John Graham

**Affiliations:** School of Biomolecular Sciences, Liverpool John Moores University, UK

## Abstract

Human peripheral blood monocytes are isolated by flotation from whole blood through a single low-density barrier prepared from OptiPrep™ at 4°C. The separation from lymphocytes depends on the more rapid rate of flotation of the monocytes because of their slightly lower density and larger size. The method works optimally only with fresh (within 2 h of drawing) EDTA-anticoagulated blood. Preliminary evidence suggests that this technique may be applicable to blood from rats.

**METHOD TYPE:** extraction, isolation, purification and separation

**SUB METHOD TYPE:** centrifugation

## INTRODUCTION

Monocytes in human peripheral blood, account for, on average, about 8% of the leucocyte population. They tend to be larger (15–20 µm) than lymphocytes (6–20 µm) and they also have a slightly lower density (Fig. 1).

A method developed by Graziani-Bowering et al.[1] permits the separation of monocytes from lymphocytes on the basis of rate of flotation from a human leucocyte-rich plasma (LRP). The method provides highly purified and viable monocytes[1] and it is described in a companion Protocol Article[2]. However because of the requirement to prepare a buffy-coat fraction from whole blood, the yield of monocytes is always compromised by the inevitable loss of leucocytes

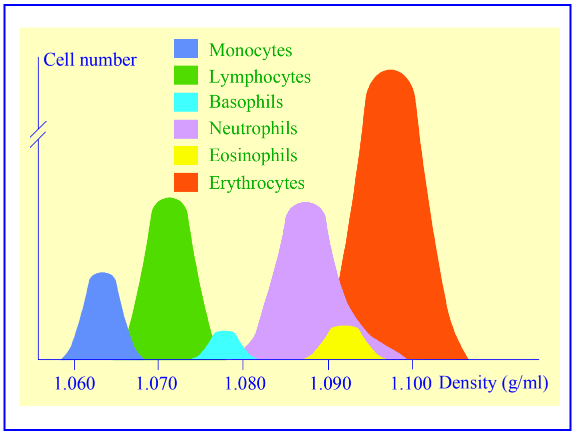

**FIGURE 1**. Density of human blood cells.

which occurs during the preparation of such a fraction. This step also adds to the overall time of preparation. The method also required the layering of two lower density layers on top of the density-adjusted LRP.

The efficacy of the method described in this protocol relies on the same principle of separation (i.e., the more rapid rate of flotation of monocytes compared to lymphocytes), but uses whole blood instead of LRP (i.e., a single centrifugation) and only one low-density layer. The method is carried out at 4°C to avoid the vesiculation within the cytoplasm of the monocytes which is sometimes observed when the separations are carried out at room temperature. When this occurs yields are also very low.

## MATERIALS AND EQUIPMENT

OptiPrep™
Diluent: Routine culture medium (e.g., RPMI or DMEM) containing 10% serum
Plastic screw-capped conical centrifuge tubes (12–15 ml, see Note 1)
Syringe with metal cannula or plastic Pasteur pipette for overlayering
Low-speed (temperature-controlled) centrifuge with swinging-bucket rotor

## METHOD

1. Collect 10 ml of blood using EDTA (1-mM final concentration as anticoagulant).
2. **Bring blood, all solutions, and equipment** to 4°C before use and shake the OptiPrep™ gently before removing an aliquot.
3. Prepare a 40% (w/v) iodixanol working solution (WS) by diluting 4 vol of OptiPrep™ with 2 vol of Diluent. This has a density of approx 1.217 g/ml (see Ref. [3] for more information about the preparation of density gradient solutions).
4. Prepare EITHER a 1.072 g/ml OR a 1.074 g/ml density barrier solution by diluting WS with Diluent: 2.14 ml + 5 ml or 2.27 + 5 ml, respectively (see Notes 2 and 3).
5. Mix 4.24 ml of WS with 10 ml of whole blood (see Note 4).
6. In a 15-ml centrifuge tube, layer 5 ml of one of the density barrier solutions over 5 ml of the blood, and then layer approx. 0.5 ml of Diluent on top (see Note 5).
7. Centrifuge at 700*g* in a swinging-bucket rotor for 30 min at 4°C. Do not use the brake during deceleration (see Note 6).

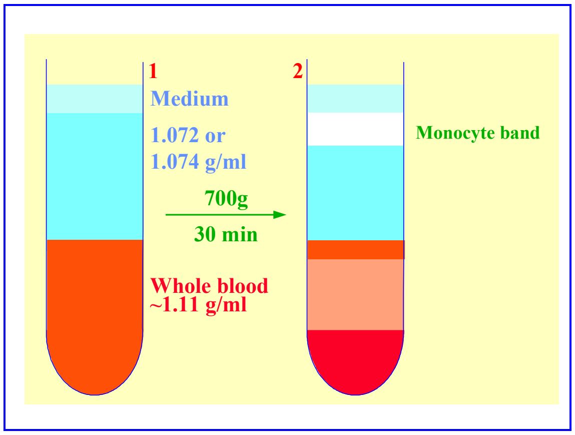

**FIGURE 2**. Separation of monocytes by flotation through a low-density barrier from whole human blood: before (1) and after (2) centrifugation.

8. Collect the monocytes, which float to the top of the 1.072 or 1.074 g/ml layer (Fig. 2).
9. Dilute the collected cells with 2 vol of Diluent and harvest by centrifugation and resuspend the pellet gently in any medium as required.

## NOTES

1. The type of centrifuge tube that is used is important as monocytes tend to adhere to some surfaces and if this occurs the cells tend to become activated. Because most purifications require the monocytes to be isolated under sterile conditions in sealed tubes, plastic tubes are obviously the tubes of choice. Some plastics are more prone to cause monocyte adhesion than others; Graziani-Bowering et al.[1] used polypropylene tubes. Because of the huge range of plastic centrifuge tubes now available commercially, it is impossible to give any firm recommendations and it is strongly advised that the tubes be checked for this problem before use.
2. The choice of density for the upper barrier depends to some extent on the operator's requirements. Use of the 1.072 g/ml barrier will give a monocyte preparation which is approx. 90% pure (as estimated by esterase staining) but the yields are approx. 40%. The higher density barrier (1.074 g/ml) will permit the recovery of more monocytes (approx. 60% of the total) but the contamination by lymphocytes is proportionately greater (approx. 85% pure).
3. For rat blood, a density for the upper layer of 1.076 g/ml is suggested.
4. The method only works satisfactorily on fresh blood (used within 2 h of drawing) from healthy individuals.
5. The uppermost layer of medium is not required for the separation process, but it avoids the banding of cells at a liquid/air interface and also prevents the cells from adhering to the walls of the tube at the meniscus.
6. If the brake is applied, mixing of the monocyte band into the lymphocytes that are located towards the bottom of the low-density barrier will occur. It is also recommended that if a slow acceleration facility is available on the centrifuge, that this should be used to maintain the stability of the interfaces.

## ACKNOWLEDGEMENTS

The author and TheScientificWorld wish to thank Axis-Shield PoC, AS, Oslo, Norway for their kind permission to adapt OptiPrep™ Application Sheet C10 in the preparation of this Protocol Article.
